# A systematic review of machine learning-based prognostic models for acute pancreatitis: Towards improving methods and reporting quality

**DOI:** 10.1371/journal.pmed.1004432

**Published:** 2025-02-24

**Authors:** Brian Critelli, Amier Hassan, Ila Lahooti, Lydia Noh, Jun Sung Park, Kathleen Tong, Ali Lahooti, Nathan Matzko, Jan Niklas Adams, Lukas Liss, Justin Quion, David Restrepo, Melica Nikahd, Stacey Culp, Adam Lacy-Hulbert, Cate Speake, James Buxbaum, Jason Bischof, Cemal Yazici, Anna Evans-Phillips, Sophie Terp, Alexandra Weissman, Darwin Conwell, Philip Hart, Mitchell Ramsey, Somashekar Krishna, Samuel Han, Erica Park, Raj Shah, Venkata Akshintala, John A. Windsor, Nikhil K. Mull, Georgios Papachristou, Leo Anthony Celi, Peter Lee

**Affiliations:** 1 Department of Gastroenterology and Hepatology, Weill Cornell Medical College, New York, New York, United States of America; 2 Department of Gastroenterology and Hepatology, Ohio State University Wexner Medical Center, Columbus, Ohio, United States of America; 3 Northeast Ohio Medical School, Rootstown, Ohio, United States of America; 4 Department of Process and Data Science, Rheinisch-Westfälische Technische Hochschule Aachen University, Aachen, Germany; 5 Department of Computational Physiology, Massachusetts Institute of Technology, Cambridge, Massachusetts, United States of America; 6 Department of Bioinformatics, Ohio State University Wexner Medical Center, Columbus, Ohio, United States of America; 7 Department of Systems Immunology, Benaroya Research Institute at Virginia Mason, Seattle, Washington, United States of America; 8 Department of Interventional Immunology, Benaroya Research Institute at Virginia Mason, Seattle, Washington, United States of America; 9 Department of Gastroenterology, University of Southern California, Los Angeles, California, United States of America; 10 Department of Emergency Medicine, Ohio State University Wexner Medical Center, Columbus, Ohio, United States of America; 11 Department of Gastroenterology, University of Illinois at Chicago, Chicago, Illinois, United States of America; 12 Department of Gastroenterology, University of Pittsburgh Medical Center, Pittsburgh, Pennsylvania, United States of America; 13 Department of Emergency Medicine, University of Southern California, Los Angeles, California, United States of America; 14 Department of Emergency Medicine, University of Pittsburgh Medical Center, Pittsburgh, Pennsylvania, United States of America; 15 Department of Medicine, University of Kentucky, Lexington, Kentucky, United States of America; 16 Department of Gastroenterology, Johns Hopkins Medical Center, Baltimore, Maryland, United States of America; 17 Department of Surgical and Translational Research Centre, University of Auckland, Auckland, New Zealand; 18 Department of Hospital Medicine and Penn Medicine Center for Evidence-based Practice, University of Pennsylvania, Philadelphia, Pennsylvania, United States of America; 19 Department of Critical Care, Beth Israel Medical Center, Boston, Massachusetts, United States of America; University of Texas Southwestern Medical Center, UNITED STATES OF AMERICA

## Abstract

**Background:**

An accurate prognostic tool is essential to aid clinical decision-making (e.g., patient triage) and to advance personalized medicine. However, such a prognostic tool is lacking for acute pancreatitis (AP). Increasingly machine learning (ML) techniques are being used to develop high-performing prognostic models in AP. However, **methodologic and reporting quality has received little attention**. High-quality reporting and study methodology are critical for model validity, reproducibility, and clinical implementation. In collaboration with content experts in ML methodology, we performed a systematic review critically appraising the quality of methodology and reporting of recently published ML AP prognostic models.

**Methods/findings:**

Using a validated search strategy, we identified ML AP studies from the databases MEDLINE and EMBASE published between January 2021 and December 2023. We also searched pre-print servers medRxiv, bioRxiv, and arXiv for pre-prints registered between January 2021 and December 2023. Eligibility criteria included all retrospective or prospective studies that developed or validated new or existing ML models in patients with AP that predicted an outcome following an episode of AP. Meta-analysis was considered if there was homogeneity in the study design and in the type of outcome predicted. For risk of bias (ROB) assessment, we used the Prediction Model Risk of Bias Assessment Tool. Quality of reporting was assessed using the Transparent Reporting of a Multivariable Prediction Model of Individual Prognosis or Diagnosis—Artificial Intelligence (TRIPOD+AI) statement that defines standards for 27 items that should be reported in publications using ML prognostic models. The search strategy identified 6,480 publications of which 30 met the eligibility criteria. Studies originated from China (22), the United States (4), and other (4). All 30 studies developed a new ML model and none sought to validate an existing ML model, producing a total of 39 new ML models. AP severity (23/39) or mortality (6/39) were the most common outcomes predicted. The mean area under the curve for all models and endpoints was 0.91 (SD 0.08). The ROB was high for at least one domain in all 39 models, particularly for the analysis domain (37/39 models). Steps were not taken to minimize over-optimistic model performance in 27/39 models. Due to heterogeneity in the study design and in how the outcomes were defined and determined, meta-analysis was not performed. Studies reported on only 15/27 items from TRIPOD+AI standards, with only 7/30 justifying sample size and 13/30 assessing data quality. Other reporting deficiencies included omissions regarding human–AI interaction (28/30), handling low-quality or incomplete data in practice (27/30), sharing analytical codes (25/30), study protocols (25/30), and reporting source data (19/30).

**Conclusions:**

There are significant deficiencies in the methodology and reporting of recently published ML based prognostic models in AP patients. These undermine the validity, reproducibility, and implementation of these prognostic models despite their promise of superior predictive accuracy.

**Registration:**

Research Registry (reviewregistry1727)

## Author summary

**Why was this study done?:**

- Acute pancreatitis is a common gastrointestinal disorder that affects a million patients worldwide annually and has an extremely variable disease course.
- Using artificial intelligence (AI), many researchers have developed prediction tools to help forecast acute pancreatitis (AP) patients’ course (e.g., tool to predict who will develop a more severe form of disease).
- Before using AI-based prediction tools to care for patients, it is extremely important to appraise the study design and conduct of the studies that produced the tools to ensure that the tools will not lead to misleading results and worse yet, widen health disparities.

**What did the researchers do and find?:**

- We conducted a comprehensive assessment of all the published and unpublished AI models for AP patients in the past 3 years by searching all the major medical databases.
- We focused on appraising the quality of the methods and whether the model-building project was transparently reported from start to finish, using established quality standards.
- Unfortunately, almost all studies omitted taking steps to ensure that AI model will not discriminate against certain populations.
- Most studies did not take steps to avoid misleadingly optimistic results.
- Many studies did not provide enough information about how the prediction tool was built, their research methods. For example, only 15 out of 27 key reporting items were included in the paper.

**What do these findings mean?:**

- The AI prediction tools used to predict outcomes for AP patients have significant flaws in how they are designed and reported so they are not ready to be implemented into clinical care.
- Concerted efforts are needed to improve the quality of the study design, conduct of the study, and reporting of the studies so clinically useful and accurate AI prediction tools can be built and used.
- Patient and stakeholder engagement to inform study design and interdisciplinary collaboration between data scientists, clinicians, and content experts are top priorities for future research in this area.

## Abstract 3

Brian Critelli, Amier Hassan and colleagues systematically review studies published in 2021-2023 that use machine learning to develop prognostic models in acute pancreatitis and critically appraise the quality of methodology and reporting therein.

## Introduction

Defined as acute inflammation of the pancreas, acute pancreatitis (AP) remains a common and costly cause of gastrointestinal-related hospitalization, with 1 million new cases each year globally and increasing incidence [1,2]. The etiology of the disease varies across patient demographics, with gallstones and alcohol comprising the majority of adult cases and diverse factors such as hypertriglyceridemia, drugs, infections, or trauma leading to a minority of cases [3]. The severity of AP can be further categorized as mild, moderately severe, or severe, with severe AP being defined by the presence of persistent organ failure [4]. The combination of persistent organ failure and infected pancreatic necrosis defines a ‘critical’ category of AP severity with the highest morbidity and mortality risk [5,6]. Survivors of AP can suffer from long-term sequelae including diabetes mellitus, recurrent or chronic pancreatitis, and pancreatic exocrine insufficiency [3,7–10]. Given the significant short- and long-term morbidity and mortality associated with AP, since 2018, and as recently as July 2024, the National Institute of Health has called for an accurate prognostic model in AP for use in research and the clinical setting [11–13]. Benefits of an accurate prognostic model are many, including enablement of cost-efficient clinical trials through cohort enrichment [14,15], identification of subphenotypes within a cohort that require different treatment strategies [16,17], and prompt triaging of patients in the emergency room [18].

Current prognostic models for AP were developed using regression-based techniques (e.g., Glasgow Criteria, Bedside Index for Severity in Acute Pancreatitis, etc.) which demonstrate suboptimal performance and limited clinical usefulness [19]. For example, in a prospective external evaluation of regression-based models predicting mortality, none of the models tested produced a post-test probability higher than 14% when “positive” [20]. There has been a call for new approaches to improve prediction accuracy [19,21]. Advances in the subset of artificial intelligence (AI) known as machine learning (ML) have facilitated the development of non-regression prediction models, which offer advantages over regression-based models by performing better in diseases with non-linear predictor–outcome relationships such as AP [22]. There has been an increasing number of published ML-based prognostic models that appear to outperform regression-based models [23–25]. However, ML experts have cited concerns regarding methodologic quality, model building practices, and lack of transparent reporting [26–28]. While there continues to be efforts to examine accuracies and clinical utility of published prognostic models in AP [19], collaborations with ML methodologists and focus on methodological and reporting qualities have been lacking. In a collaborative effort between content experts, clinicians, and methodologists, we therefore undertook a systematic review and critical appraisal of recent published studies proposing new non-regression ML-based prognostic models to detail any methodological shortcomings and/or gaps in reporting.

## Methods

Detailed methodology of this review has been published elsewhere [29]. We conducted a systematic review of all studies published between January 2021 and December 2023 in which a non-regression, ML-based prognostic model in AP was developed and/or validated (either internally or externally), with or without model updating. This review included studies of prospective or retrospective design including post-hoc analysis of clinical trials that: (a) enrolled only adult patients (i.e., 18-years old or older), (b) contained a prognostic model of AP developed with non-regression ML technique(s), (c) predicted any outcome(s) of AP, and (d) published in English. Studies involving participants with chronic pancreatitis, pancreatic cancer, or post-surgical pancreatitis were excluded, as were studies with animals, regression-based models, or models that predict the development of AP instead of disease outcomes. Studies published in abstract form only and review articles were also excluded.

We searched the databases MEDLINE (OvidSP) and EMBASE (OvidSP) from January 1, 2021 to December 31, 2023 (Date of search for all data sources, January 31). We also searched pre-print servers medRxiv, bioRxiv, and arXiv for pre-prints registered between January 2021 and December 2023. Our search was limited to the most recent 3 years for the following reasons. (1) Significant advancements in AP management paradigm has led to a significant change in the natural history/prognosis of the disease over the last decade [30–37]. It was important to identify models trained/evaluated on datasets generated from the most recent cohort of AP. (2) New algorithms rapidly emerge, replacing older algorithms, and temporal quality degradation is an established phenomenon in AI models [38]. Validated search strategies [39,40] were used and are listed in S1 and S2 Tables, respectively. For medRxiv, bioRxiv, and arXiv, the search term “acute pancreatitis” to maximize the sensitivity of our search. Covidence software (Melbourne, Australia) was used to screened title-abstract and full text in sequential steps. Each stage required concordance between two independent reviewers (LN, IL, KT, JP, AH, BC, NM, or AL). Disagreements were resolved by a third independent reviewer (PJL or LAC). Included studies were then appraised in terms of risk of bias (ROB) in study design, completeness of reporting, and for summarization of model predictive performances. Necessary data for Prediction Model Risk of Bias Assessment Tool (PROBAST) and Transparent Reporting of a Multivariable Prediction Model of Individual Prognosis or Diagnosis—Artificial Intelligence (TRIPOD+AI) evaluation were extracted in accordance with the Critical Appraisal and Data Extraction for Systematic Reviews of Prediction Modelling Studies (CHARMS) checklist [41].

### Methodologic quality assessment

The PROBAST was used to assess both ROB in study design of prospective models across four main domains: participants, predictors, outcomes, and analysis [42]. The assessment of Applicability section of PROBAST was planned if metadata were appropriate and feasible for meta-analysis. To optimize the validity of the PROBAST assessment, all evaluators underwent PROBAST rater training, which entailed weekly meetings with an AP content expert trained by PROBAST developers (PJL) to review all 20 signaling questions. Data scientists (JNA, LL, JQ, or DR) and ML content experts (LAC) were engaged to accurately complete CHARMS and PROBAST. Each model was assessed via the PROBAST framework by two independent reviewers (LN, IL, KT, JP, AH, BC, NM, AL, JNA, LL, JQ, or DR), and disagreements were resolved by an independent third reviewer (PJL or LAC). The pair of reviewers comprised a clinician and a data scientist. The ROB in each domain and overall ROB were reported for all studies.

### Reporting quality assessment

To assess the quality of the reporting, we decided to use TRIPOD+AI statement, which contains a comprehensive list of items that need to be reported for papers reporting development and/or validation of prognostic AI model [43]. List of sections and items on this list covers every key part of a manuscript including title, abstract, introduction, methods, results, and discussion. Additionally, it contains items related to open science and patient and public involvement. Summary statistics of quality of reporting according to the standards of TRIPOD+AI [43] were calculated for each study. This review has been registered at Research Registry (reviewregistry1727). All data reporting in this systematic review adhered to the guidelines of Preferred Reporting Items for Systematic reviews and Meta-Analyses (PRISMA) and the checklist can be found in a separate supplementary file (S1 PRISMA Checklist).

## Results

The results data are publicly available at https://doi.org/10.6084/m9.figshare.26078743.v1. Our search strategy identified 6480 studies published between January 2021 and December 2023, of which 30 met eligibility criteria (S1 Fig). Studies originated from China (22), the United States (4), Hungary (2), Turkey (1), and New Zealand (1) (Table 1).

**Table 1:** Basic characteristics of included studies.

Author	Publication year	Study site	Type of study	Number of centers	Number of participants	Racial category	Machine learning algorithms	AUC	Type of predictors included *	Outcome predicted^†^
Ding [44]	2021	United States of America	Retrospective cohort	1	337	Reported	Neural Network (incl. deep learning)	0.77	1, 2, 3, 4	3
Jin [45]	2021	China	Retrospective cohort	1	369	NR	Neural Network (incl. deep learning)	0.98	4	5
Langmead [24]	2021	United States of America	Secondary analysis of prospective cohort study designed for another reason	1	133	Reported	Tree-based models	0.91	5	1
Xu [46]	2021	China	Retrospective cohort	3	447	NR	Other (Adaptive Boost)	0.83	4, 5	10
Zhu [47]	2021	China	Retrospective cohort	6	711	NR	Tree-based models Neural network	0.99	1, 3, 4	13
Hameed [48]	2022	United States of America	Administrative database	2	6,326	NR	Tree-based models	0.94	1, 4	3
Hong [49]	2022	China	Retrospective cohort	1	648	NR	Tree-based models	0.96	1, 3, 4	1
İnce [50]	2022	Turkey	Retrospective cohort	1	1,334	NR	Other (Gradient Boost)	0.91–0.98	1, 3, 4	1,3,4
Kimita [51]	2022	New Zealand	Prospective cohort	1	160	Reported	Tree-based models	0.67	5	6
Kiss [52]	2022	Hungary	Prospective cohort	30	2,387	NR	Tree-based models	0.76	1, 4	7
Kui [53]	2022	Hungary	Prospective cohort	28	1,184	NR	K-nearest neighbor	0.81	1, 2, 4	1
Li [54]	2022	China	Prospective cohort	7	915	NR	Tree-based models Support vector machine Random Forest LightGBM Ensemble	0.79–0.90	1, 2, 4	2, 3, 4, 8, 9
Shi [55]	2022	China	Retrospective cohort	3	2,846	NR	Tree-based models	0.90, 0.98	1, 4	3, 5
Thapa [56]	2022	United States of America	Administrative database	700	371,885	Reported	Tree-based models	0.92	1, 2, 4	1
Yan [57]	2022	China	Retrospective cohort	1	151	NR	Tree-based models	NR	2, 4	3
Yang, D [58]	2022	China	Retrospective cohort	1	996	NR	Tree-based models Neural Network (incl. deep learning) XGBoost	0.73–0.91	1, 2, 4	5
Yang, Y [59]	2022	China	Retrospective cohort	2	424	NR	Tree-based models	0.91	1, 3, 4, 5	5
Yin [60]	2022	China	Retrospective cohort	3	1,012	NR	Tree-based models Gradient Boosting Machines Neural Networks XGBoost	0.87–0.95	1, 3, 4	1
Yuan [61]	2022	China	Retrospective cohort	2	5,280	NR	Tree-based models	0.87	1, 2, 3, 4	4
Zhou [62]	2022	China	Retrospective cohort	1	441	NR	XGBoost	0.91	1, 3, 4	1
Chen [63]	2023	China	Retrospective cohort	1	978	NR	Neural Network (incl. deep learning)	0.82, 0.92	2, 3, 4	1, 2
Liang [64]	2023	China	Administrative database	1	1,798	NR	Neural Network (incl. deep learning)	0.98	3	2, 5
Luo, Z [65]	2023	China	Retrospective cohort	2	673	NR	Naive Bayes	0.96	1, 3, 4	1
Luo, J [66]	2023	China	Retrospective cohort	1	13,645	NR	Neural Network (incl. deep learning)	0.91	2, 4	10
Ren [67]	2023	China	Retrospective cohort	1	531	NR	Tree-based models	0.81	1, 3,4	11
Yang, D [68]	2023	China	Retrospective cohort	1	292	NR	Tree-based models*	0.995	4, 5	5
Zhang, W [69]	2023	China	Retrospective cohort	1	440	NR	Tree-based models	0.93	1, 3, 4	5
Zhang, J [70]	2023	China	Retrospective cohort	4	820	NR	CatBoost Random Forest Neural Network	0.52–0.75	1, 4	12
Zhang, M [71]	2023	China	Retrospective cohort	1	460	NR	Bayesian Support Vector Machine Ensembles of Decision Tree	0.81–0.89	4	5
Zhao [72]	2023	China	Retrospective cohort	1	215	NR	Tree-based models	0.89	3	2

*Type of predictors included: 1 =  clinical history (incl. demographics, social, medical history), 2 =  physical exam findings, 3 =  radiologic features, 4 =  laboratory values, 5 =  cytokines/new biomarker.

^†^Outcome(s) predicted: 1 =  severe pancreatitis, 2 =  mild acute pancreatitis, 3 =  mortality (all-cause, acute pancreatitis specific, does not specify), 4 =  intensive care unit admission, 5 =  moderately severe and severe pancreatitis, 6 = other, 7 =  pancreatic necrosis, 8 =  length of stay, 9 =  pancreatic necrosis – infected, 10 =  multisystem organ dysfunction/failure, 11 =  recurrent pancreatitis, 12 = new onset diabetes, 13 = Intra-abdominal infection.

**Fig 1:**
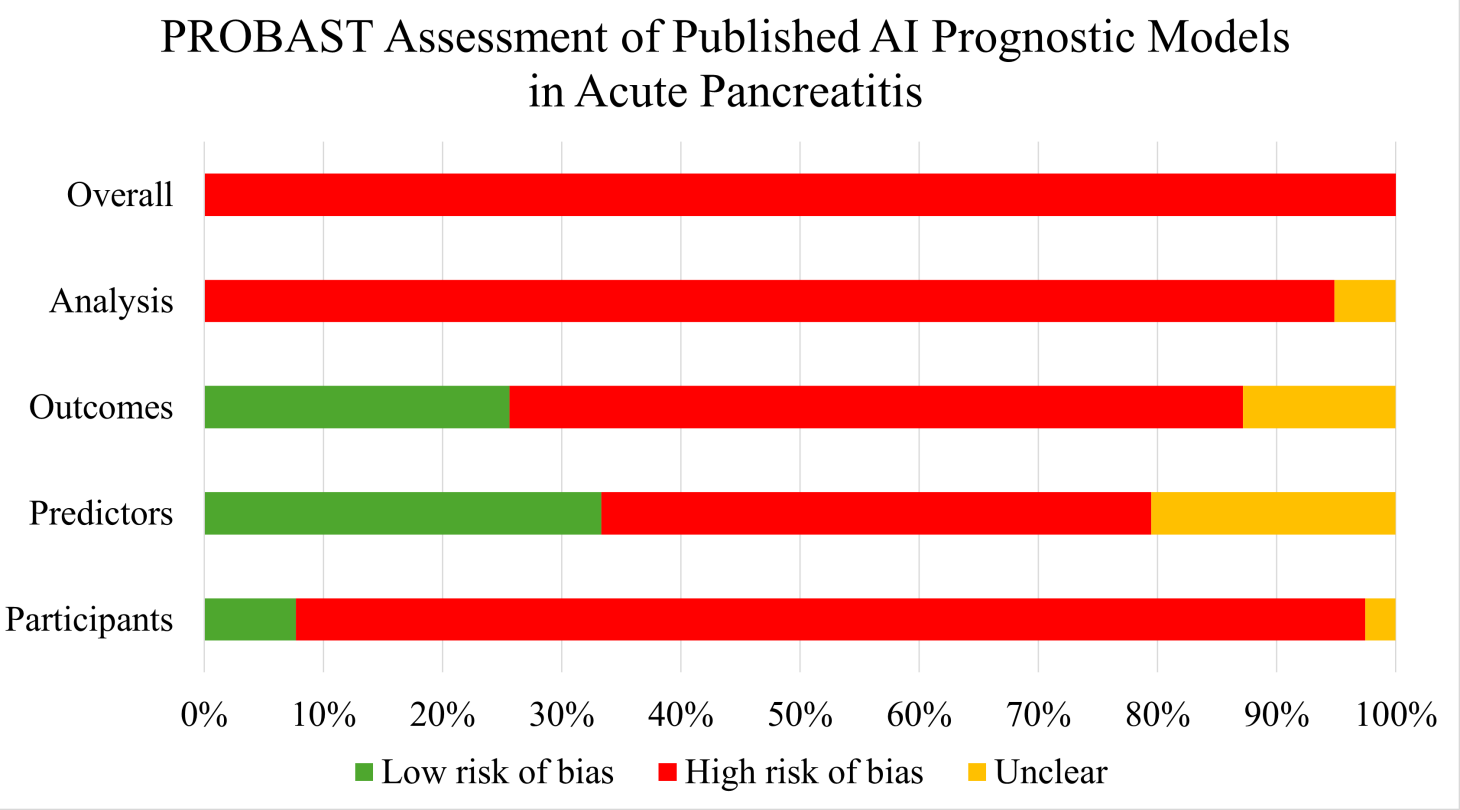
Summary of risk of bias in four domains assessed by PROBAST.

**Fig 2:**
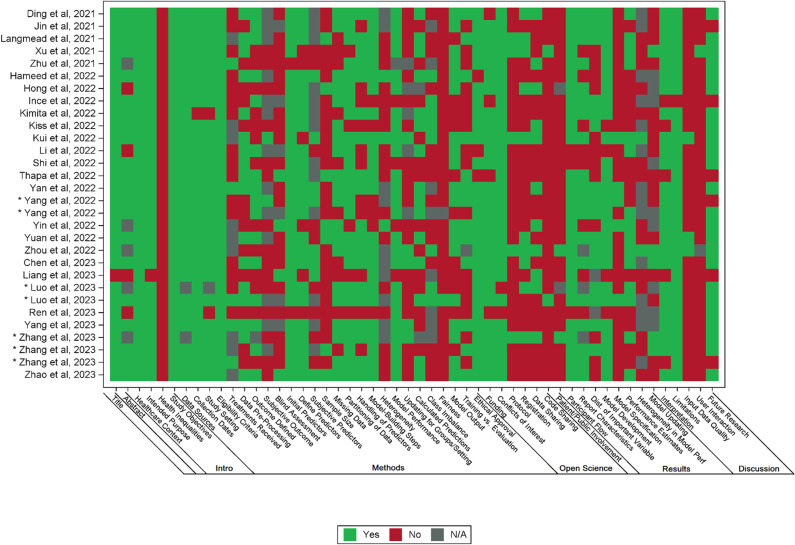
Heatmap depicting common areas of deficiencies in reporting standards as assessed by TRIPOD+AI. * Publication has same first author and year as another paper listed; PMID of each *  in ascending order: Yang and colleagues (2022): 35430680, 35607360 [58,59]. Luo and colleagues (2023): 36653317, 36773821 [65,66]. Zhang and colleagues (2023): 36902504, 36964219, 37196588 [69–71].

All 30 studies reported the development of a new ML-based prognostic model, but only one study included external validation step of the newly developed model. Nearly three-fourths (22/30) of included studies were retrospective cohort, while only five studies were prospective, of which one was a secondary analysis and three studies used administrative databases. Five studies developed more than one model, resulting in a total of 39 models developed in 30 studies. The most common ML algorithms were tree-based models (20/39) and neural networks (7/39). AP severity (21/39) or mortality (6/39) were the most common outcomes predicted. The most common methods of internal validation were cross-validation (23/39) and bootstrapping (17/39). For 31/39 models, shrinkage methods were not used to evaluate for or adjust for optimism (shrinkage methods: techniques used to account for magnitude of noise in the dataset contributing to overinflation of predictive performance). A summary of pertinent descriptive statistics collected as per the CHARMS checklist is provided in Table 1. Six studies developed more than one ML-model using the same dataset, presenting the parameters of the “best performing” model (Table 1). Every model had at least one domain in which the ROB was classified as high (Fig 1), meaning that all 39 models were assessed to be at high ROB by PROBAST standards (see S3 Table for individual model’s ROB rating). The median number of TRIPOD+AI items that were reported on in the 30 studies was 15/27 (range 6–20). Ren and colleagues reported the least number of items, whereas Kui reported on the greatest number of items [52,57]. No study reported on all the items. A comprehensive breakdown of the number of TRIPOD+AI items reported on in each study is given in S4 Table and on the heatmap for visual presentation of the data (Fig 2). The primary model performance metric in the 39 included studies was the area under the curve (AUC), with a mean AUC of 0.91 (SD 0.08) for all included models.

### Risk of bias in four domains of methodology as assessed by PROBAST

PROBAST ratings of the 39 models based on individual studies are summarized in S3 Table. Assessment of Applicability was not applicable to the objectives of this review. As the primary objective was to assess the methodologic quality and because of marked heterogeneity of the cohorts and the different definitions and determination of outcomes, a synthesis of the meta-data was not undertaken.

#### Participants domain.

In this domain, there was a high ROB with 35/39 models. The data source was not appropriate with 31/39 models. The inclusions and exclusions of participants was not appropriate in 26/39 models.

#### Predictors domain.

In this domain, there was a high ROB with 18/39 models. The predictors were not defined and measured in a similar way for all participants in 12/39 models. Assessor blinding to the outcome data was not done with 30/39 models. In 8/39 studies predictors were included when the result would not be available at the time of applying the prognostic model.

#### Outcomes domain.

In this domain, there was a high ROB with 24/39 models. While outcomes were *defined* in a standard way in 33/39 models, they were not *determined* appropriately in 20/39 models. The way that outcomes were determined was not reported for 1/39 models [58]. Outcomes were not defined and determined in a similar way in 13/39 models. Blinding was not performed in 24/39 models. Outcomes were included as predictors in 17/39 models.

#### Analysis domain.

In this domain, there was a high ROB with 37/39 models (Fig 1). The common deficiencies in this domain were no accounting for overfitting and optimism (i.e., no shrinkage methods employed) in 31/39 models, none or inappropriate reporting of data complexity in 38/39 models (Fig 2), insufficient sample size in 28/39 models, and selection of predictors relied solely on univariate analysis in 26/39 models.

### Quality of reporting as assessed by TRIPOD+AI

#### Title, abstract, introduction section.

All 30 studies reported to the standards of TRIPOD+AI except in one important sub-item. No study reported the health inequalities that may exist in outcomes between sociodemographic groups (Fig 2 and S4 Table).

#### Methods section.

Twenty-eight studies described the sources of data, study dates, setting and eligibility were described in 28/30 studies but only 5/30 studies reported details of any treatment received where treatment might have influenced the outcome of interest. Other frequent omissions included no description of model fairness and their rationale (28/30), no sample size justification (23/30), no blinding of assessors (20/30), no reporting differences between training and evaluation data (16/30), no outcome measurement (15/30), no description of data preparation and pre-processing (13/30), no reporting of elements pertinent to outcome definition (13/30), and no assessment of study quality (13/30).

#### Open science and patient/public involvement section.

There was no reporting on whether a protocol was prepared, available or accessed in 25/30 studies. There was no report as to the availability of study data (9/30) or analytical code (28/30). There was comment on whether patients and public were involved in 26/30 studies.

#### Results section.

There was insufficient detail of the prediction model to allow external validation in 25/30 studies. Reporting details of the prediction model performance in key subgroups (e.g., sociodemographic) was not available in 15/30 studies.

#### Discussion section.

Items pertaining to the usability of the model in the context of current care were usually not discussed. Only 3/30 studies described how poor quality or missing data should be handled with clinical implementation of the model. Only 1/30 study specified whether users will be required to interact with handling of the input data or use of the model and what level of expertise is required to use the model.

#### Percentage of fidelity to TRIPOD+AI.

To investigate if TRIPOD+AI fidelity correlated with study origin, design, outcomes or ROB, the studies were grouped into four quartiles. The top quartile group contained studies with the highest fidelity percentage and bottom quartile with the lowest fidelity percentage. The degree of fidelity percentage did not appear to correlate with any particular study origin, type, number of centers, outcomes or ROB (S5 Table).

#### Rationale against performing subgroup analyses.

Even though several of the included studies developed models predicting similar outcomes—including all-cause mortality [45,46,48,53,58,61], severe pancreatitis [24,44,47,48,52,55,59,65,71], and ICU admission [48,53,66], as examples—the decision was made not to perform subgroup analyses stratified by similar endpoints. All but one model was judged to have high ROB in at least two out of the four PROBAST domains and none of the models were at low ROB in the statistical analyses domain. Other subgroup analyses were not performed as meaningful discoveries or different conclusions were unlikely given the high ROB across all studies.

## Discussion

In this systematic review, we assessed the quality of the methodology and reporting of studies that develop and/or validated non-regression ML-based models in AP literature. While the performance of the published models was high (mean AUC 0.91), we identified several key limitations in the recently published models. Unfortunately, these shortcomings are like those identified in other fields such as oncology [28] and anesthesiology [73]. First, the concern relates to the high ROB most notably in the statistical analysis section, which can undermine the validity of the models. Second, due to the lack of external validation studies, generalizability of the ML models may be limited. Third relates to open science practice, where in over 90% of the studies, the code was not shared and no information was provided on how the model was built. Additionally, there was a lack of reporting on how the ML model can be implemented in clinical practice. Lastly, none of the studies described potential health inequities among different sociodemographic groups, which risks widening disparities in healthcare, if implemented in real clinical practice.

The quality of the statistical analyses is one of the most important facets of model development. The PROBAST ROB tool dedicates nine signaling questions to this domain [42]. Two particularly deficient areas were sample size justification and guarding against overfitting. A robust sample size (especially for a ML model) and guarding against overfitting are critically important. When these steps are omitted, a model may perform well in the development dataset, but the predictive performance may not be reproducible [74]. We found that most published studies developed a model with a sample size of less than 1,000 participants and median events per variable was 9.5. Even for regression-based models, the minimum recommended events per variable is 20 [42]. While events per variable is not a singular reflection of sufficient sample size, it is generally accepted that ML models require much larger sample size (than regression-based models) due to the risk of model instability [75].

Potentially limited generalizability of the published models needs to be highlighted. Only one study conducted external validation but with limitations [60], and all but five studies were single-center design. While AP is a common gastrointestinal disease, with an annual worldwide 1 million new cases a year [76], international or large multi-center consortiums with efforts to build a generalizable model have been lacking. Lack of such collaboration results in siloed attempts at building models that may not be clinically utilized due to poor reproducibility and generalizability. As with the case with the regression-based models [21], we are seeing a similar trend in ML-based models in AP.

Ultimately, prognostic models are built to aid clinical decision-making or enhance cohort enrichment in a research study. Therefore, steps need to be taken to thoughtfully consider real-life issues we will face when trying to deploy these models (e.g., ways to deal with missing values in real clinical practice when patients won’t have the data elements necessary for the ML model). We also found key missing items relevant to open science, that limit external validation studies by other investigators and clinical implementation by the hospitals. For example, only five studies shared the code to permit third-party evaluation and implementation [52,56,61,62,65], only three studies gave guidance on how to handle missing data [52,66,71], and one study detailed the specifics of what constitutes human–AI interaction [60]. As important, aspects of model building relevant healthcare equity (e.g., comparison of performance estimates among different sociodemographic subgroups) were not evaluated. Such deficiency leads to a potential to produce a model that widens the socioeconomic disparities [77].

Our study has several strengths. For transparency and rigor of our methodology, we have published our methods and adhered strictly to the standards of TRIPOD-SR/MA. Our work was conducted in collaboration between data scientists, ML methodologist, and content experts in AP, which we believe enhances the reliability of our findings. There are multiple aspects to PROBAST and TRIPOD+AI assessment that require both AP content and ML methodology expertise. Moreover, rigorous internal training for PROBAST assessment preceded the project, enhancing the validity of our ROS assessment.

Several limitations deserve mention. Our search strategy extended to only the last 3 years so it is possible that our findings may not be fully representative of all the ML models published for AP thus far. We also recognize that TRIPOD+AI was published in 2024. While the pre-print version of TRIPOD+AI has been available since 2021, it is important to recognize that many investigators will not have been aware of the document. However, this does not diminish the importance of the message of our study and further highlight why TRIPOD+AI needed to be published. Additionally, TRIPOD+AI encapsulates many sound reporting practices that were already well established with its previous version, the TRIPOD statement published in 2015 [78]. Second, while PROBAST was developed by expert methodologists, it is possible that models deemed high ROB by PROBAST may still be valid, reproducible, and generalizable in AP. However, there is emerging data from other diseases that suggest models deemed high ROB by PROBAST perform poorly external validation studies [79]. Furthermore, due to significant study design heterogeneity, we did not perform quantitative analyses so quantitative conclusions are lacking. It is worth noting that the outcomes predicted were overwhelmingly severity-related in the included studies. While predicting severity is important for early inpatient management, additional clinically meaningful outcomes could be considered in the future. These include patient reported outcomes such as quality of life, disability or unemployment after discharge, and outcomes relevant to invasive intervention planning (e.g., a model to predict complication-free resolution of pancreatic necrosis). By focusing on these intermediate and long-term outcomes, the strengths and advantages of ML-based technique (i.e., imaging pattern recognition and analyses of complex multi-dimensional data) can be leveraged to forecast an accurate prognosis which would otherwise be challenging with regression-based models. For example, candidate covariates in such a model could include the extent of pancreatic and extra-pancreatic injury as determined by imaging studies, sets of laboratory values, social determinants of health, and comorbidities. These then could be included in a ML-based model to predict the suggested outcomes mentioned above, or be used to create subphenotypes of patients who survive AP

In conclusion, the potential benefit of ML-based prognostic models is evident with an overall high AUC (mean 0.91 ± 0.8SD). However, this study indicates that there should be great caution in implementing the reported models because of the major concerns with the quality of the methodology and reporting. These raise questions about the validity, reproducibility, and generalizability of the prognostic models. It is recommended that AP-specific, standardized methodology that covers all four PROBAST domains and all items within TRIPOD+AI be used in developing and validating ML-based prognostic models. Only then implementation should be considered. Our study findings provide valuable baseline assessment of the quality of methods and reporting of ML-based models in AP. It is also timely given the recent publication of TRIPOD+AI [43], which was published in January of 2024 as an expansion of the original TRIPOD checklist released in 2015 [78]. TRIPOD provided reporting recommendations for prediction model studies and was subsequently adjusted for subsets of prediction model studies (i.e., TRIPOD for abstracts, TRIPOD-Cluster for models with clustered data, TRIPOD-SRMA systematic reviews and meta-analyses, TRIPOD-P for study protocols, and TRIPOD+AI for models with ML methods) [43]. Thus, our study provides an assessment of the landscape of quality of reporting in the AP literature, and hopefully will draw attention to these important facets of conducting and reporting prognostic model studies. It is beyond the scope of this study to provide an exhaustive set of recommendations on how to improve the methods and reporting of AP prognostic model studies. Nevertheless, informed by our study, we have listed high-priority areas of improvement and our suggestions for investigators, journal editors and reviewers in Table 2.

**Table 2:** High-priority areas in methodology and reporting that could be improved.

Methodology	
High Priority Areas for improvement:	Suggestions for investigators
Patient and end-user engagement in study design and outcome measures	Engage patients and intended end-users of the model (e.g., providers in the emergency room, medical floors, intensive care unit etc.) for patient-centered and clinically useful models. For example, prediction of length of stay (instead of severity) may be the most useful endpoint for emergency room providers given the pressure for bedspace in the emergency department. Patients may care more about predicting the probable length of disability attributable to disease more than whether they have mild disease or not.
Standardization of methodology where appropriate	Consider standardizing the definitions and methods by which common covariates and outcomes are determined. This promotes study design homogeneity, enhances reproducibility, and valid meta-data synthesis. For example, parenchymal injury could be *defined* according to Computed Tomography Severity Index and to be *determined* by radiologists with at least 5 years of experience.
Impact of model implementation on outcomes	In addition to assessing models’ performance, study design needs plans and steps to evaluate models for their impact on patient outcomes. For example, impact of a prognostic model in reducing unplanned readmissions from alcoholic pancreatitis by accurately selecting patients who will benefit from intensive alcohol cessation intervention.
Advanced planning for practical implementation	There needs to be a strategy for handling missing values during the real-time implementation of the model. For example, strategy for situations where important features are missing.
Statistical analyses planning	It is generally agreed that prediction models based on machine learning methods require extremely large datasets (e.g., could need more than 10 times as many events for each predictor than regression-based models) [80] Estimate required sample size based on published guidelines for regression-based models. If resource constraints do not permit even the sample size required for the regression-based model, refrain from developing an ML model, but consider building a regression-based model with robust steps against overfitting (e.g., penalization and shrinkage approaches) [80]. Collaboration between content experts, data scientists, and methodologists at study design inception is critical [43,81].
Health equity	Intentional enrollment of participants across all sociodemographic groups to optimize the chance for a truly representative cohort Plan a priori evaluation of model performance across different sociodemographic groups
Model evaluation	Develop collaborative consortiums to design very large datasets using standardized methodology, to permit external validation of previously developed models. Be mindful of *data drift*^∞^. If a model is clinically implemented in clinical practice, have a robust plan to recalibrate and update the model at a regular interval based on the model’s impact on patient outcomes (e.g., is the implementation of the model leading to improved outcomes?), health equity (e.g., is the model widening health disparity among different socioeconomic groups?), etc. [38,82].
**Transparent Reporting**	
Areas for improvement:	Implications for investigators
Open science practices	Report registration information and protocol to allow others to assess protocol fidelity. Share full details of the model (including data and code) and its development to enable assessment of reproducibility and permit external validation.
Real-life clinical applicability considerations	Specific details on how to optimize model’s integration into clinical workflow (e.g., specific details on how to handle missing values when integrated into electronic health record etc.). Include details on levels of expertise required to integrate the model into clinical practice for technical information for integration (e.g., into electronic health record software) and to design user interface for implementation into clinical practice.
Health equity	Describe specific steps taken to address model’s fairness^❖^. If evaluating fairness was challenging, how could future investigators overcome the challenges. If the model was assessed to be “unfair”, what further work is required to address it
**Suggestions for Journal Editors and Reviewers**	
Assessment of study methodology	Consider requiring interdisciplinary collaboration with AI data scientist as a prerequisite for submission and publication. Consider implementing a structured assessment of the risk of bias (e.g., PROBAST) in submitted manuscripts that include AI models in acute pancreatitis to aid publication decisions. For example, publication of studies that have high risk bias across all domains could be discouraged.
Assessment of study’s reporting practice	Consider implementing a quantitative assessment of fidelity of a submitted manuscript to standards recommended by methodologists to inform whether a manuscript should be considered for publication. For example, editors could establish non-negotiable elements as prerequisites for publication.

^∞^Data drift is defined as a systematic change in the distribution of model input parameters over time that leads to model performance decline.

^❖^Fairness is defined according to the authors of TRIPOD+AI [43]. In short, prediction models should be designed and implemented in a non-discriminatory way (i.e., does not discriminate against any group of individuals, create or worsen healthcare disparities). It also means that the model is developed, evaluated, implemented, and deployed through engaging multiple different stakeholders (e.g., patient, clinicians, public etc.)

Abbreviations: PROBAST, Prediction Model Risk of Bias Assessment Tool; TRIPOD+AI, Transparent Reporting of a Multivariable Prediction Model of Individual Prognosis or Diagnosis—Artificial Intelligence.

## Supporting information

S1 Fig PRISMA flow diagram. (DOCX)

S1 Table Search strategy in MEDLINE. (DOCX)

S2 Table Search strategy in EMBASE. (DOCX)

S3 Table PROBAST rating of models from individual studies. (DOCX)

S4 Table Responses on TRIPOD+AI and overall fidelity to transparent reporting standards for machine learning studies (*N* = 30). (DOCX)

S5 Table Division of TRIPOD+AI fidelity percentage into four quartiles. (DOCX)

S1 PRISMA Checklist PRISMA 2020 Checklist. (DOCX)

## Abbreviations

**:**
AI: artificial intelligence
AP: acute pancreatitis
AUC: area under the curve
CHARMS: Critical Appraisal and Data Extraction for Systematic Reviews of Prediction Modelling Studies
ML: machine learning
PRISMA: Preferred Reporting Items for Systematic reviews and Meta-Analyses
PROBAST: Prediction Model Risk of Bias Assessment Tool
ROB: risk of bias
TRIPOD+AI: Transparent Reporting of a Multivariable Prediction Model of Individual Prognosis or Diagnosis—Artificial Intelligence
