# Supplementary figures and images for: A systematic review of machine learning-based prognostic models for acute pancreatitis: Towards improving methods and reporting quality
Source: PLoS Med. 2025 Feb 24;22(2):e1004432. doi: 10.1371/journal.pmed.1004432 (PMC11870378; doi:10.1371/journal.pmed.1004432)

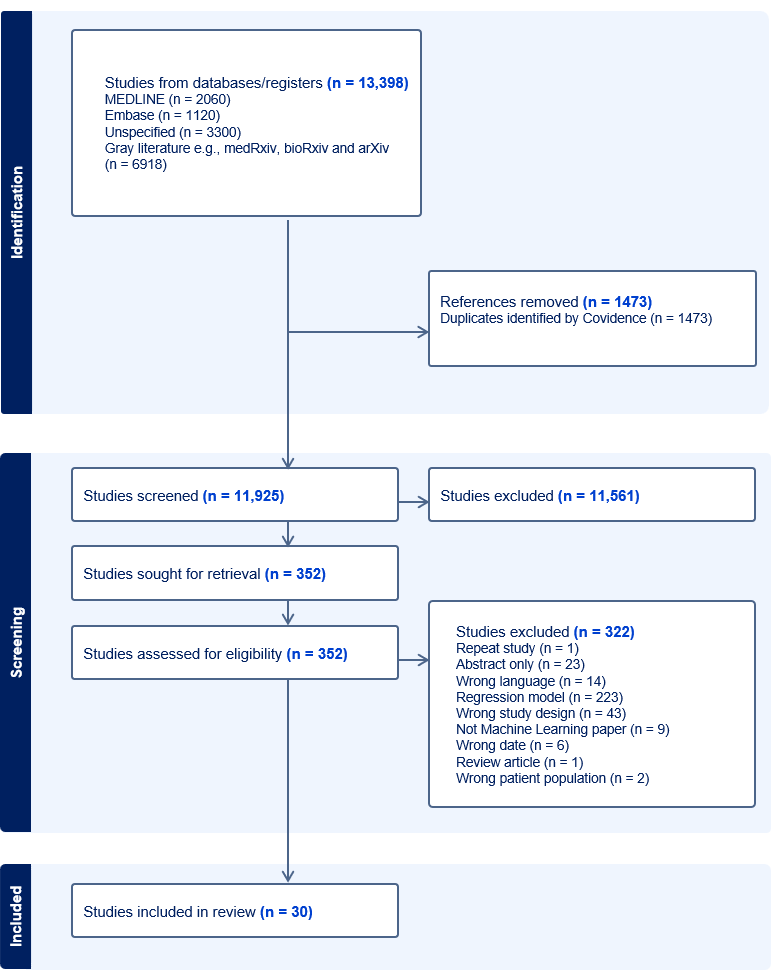

Supplement: S1 Fig — (DOCX) [file pmed.1004432.s001.docx]
