# Supplementary material for: A systematic review of machine learning-based prognostic models for acute pancreatitis: Towards improving methods and reporting quality
Source: PLoS Med. 2025 Feb 24;22(2):e1004432. doi: 10.1371/journal.pmed.1004432 (PMC11870378; doi:10.1371/journal.pmed.1004432)
Supplement: S1 Table — (DOCX) [file pmed.1004432.s002.docx]

**Supplementary Table 1:** Search Strategy in Medline

| 1. Validat$ OR Predict$.ti. OR Rule$.mp. [mp=title, book title, abstract, original title, name of substance word, subject heading word, floating sub-heading word, keyword heading word, organism supplementary concept word, protocol supplementary concept word, rare disease supplementary concept word, unique identifier, synonyms] 2. (Predict$ AND (Outcome$ OR Risk$ OR Model$)).mp. [mp=title, book title, abstract, original title, name of substance word, subject heading word, floating sub-heading word, keyword heading word, organism supplementary concept word, protocol supplementary concept word, rare disease supplementary concept word, unique identifier, synonyms] 3. ((History OR Variable$ OR Criteria OR Scor$ OR Characteristic$ OR Finding$ OR Factor$) AND (Predict$ OR Model$ OR Decision$ OR Identif$ OR Prognos$)).mp. [mp=title, book title, abstract, original title, name of substance word, subject heading word, floating sub-heading word, keyword heading word, organism supplementary concept word, protocol supplementary concept word, rare disease supplementary concept word, unique identifier, synonyms] 4. (Decision$ AND (Model$ OR Clinical$ OR Logistic Models)).mp. [mp=title, book title, abstract, original title, name of substance word, subject heading word, floating sub-heading word, keyword heading word, organism supplementary concept word, protocol supplementary concept word, rare disease supplementary concept word, unique identifier, synonyms] 5. (Prognostic AND (History OR Variable$ OR Criteria OR Scor$ OR Characteristic$ OR Finding$ OR Factor$ OR Model$)).mp. [mp=title, book title, abstract, original title, name of substance word, subject heading word, floating sub-heading word, keyword heading word, organism supplementary concept word, protocol supplementary concept word, rare disease supplementary concept word, unique identifier, synonyms] 6. (Stratification OR “ROC Curve” OR Discrimination OR Discriminate OR “c-statistic” OR “C statistic” OR “Area under the curve” OR AUC OR Calibration OR Indices OR Algorithm OR Multivariable).mp. [mp=title, book title, abstract, original title, name of substance word, subject heading word, floating sub-heading word, keyword heading word, organism supplementary concept word, protocol supplementary concept word, rare disease supplementary concept word, unique identifier, synonyms] 7. 1 or 2 or 3 or 4 or 5 or 6 8. Exp *Pancreatitis-Associated Proteins/ or exp *Pancreatitis/ or exp *Pancreatitis, Acute Hemorrhagic/ or exp *Pancreatitis, Acute Necrotizing/ or exp *Pancreatitis, Alcoholic/ 9. (Acute adj3 pancrea*).tw. 10. 8 or 9 11. 7 and 10 |
| --- |
