# Supplementary material for: A systematic review of machine learning-based prognostic models for acute pancreatitis: Towards improving methods and reporting quality
Source: PLoS Med. 2025 Feb 24;22(2):e1004432. doi: 10.1371/journal.pmed.1004432 (PMC11870378; doi:10.1371/journal.pmed.1004432)
Supplement: S2 Table — (DOCX) [file pmed.1004432.s003.docx]

| 1. exp *Pancreatitis-Associated Proteins/ or exp *Pancreatitis/ or exp *Pancreatitis, Acute Hemorrhagic/ or exp *Pancreatitis, Acute Necrotizing/ or exp *Pancreatitis, Alcoholic/  2. (Acute adj3 pancrea*).ti,ab  3. 1 or 2  4. follow-up.mp.  5. prognos*.tw.  6. ep.fs  7. 4 or 5 or 6  8. 3 and 7 |
| --- |

**Supplementary Table 2:** Search Strategy in EMBASE
