# Supplementary material for: A systematic review of machine learning-based prognostic models for acute pancreatitis: Towards improving methods and reporting quality
Source: PLoS Med. 2025 Feb 24;22(2):e1004432. doi: 10.1371/journal.pmed.1004432 (PMC11870378; doi:10.1371/journal.pmed.1004432)
Supplement: S5 Table — (DOCX) [file pmed.1004432.s006.docx]

| **Supplementary Table 5:** Division of TRIPOD+AI fidelity percentage into four quartiles | | | | |
| --- | --- | --- | --- | --- |
|  | Top quartile (highest fidelity) (n=8) | Second quartile (n=7) | Third quartile (n=8) | Bottom Quartile (n=7) |
|  | Range of % fidelity: 64.8-75.9 | Range of % fidelity: 54.6-64.7 | Range of % fidelity: 50.0-54.5 | Range of % fidelity: 22.2-49.9 |
| Country of origin, n (%) |  |  |  |  |
| China | 6 (75%) | 5 (71.4%) | 6 (75%) | 5 (71.4%) |
| Hungary | 1 (12.5%) | 0 (0%) | 0 (0%) | 1 (14.3%) |
| New Zealand | 0 (0%) | 0 (0%) | 1 (12.5%) | 0 (0%) |
| Turkey | 0 (0%) | 0 (0%) | 0 (0%) | 1 (14.3%) |
| USA | 1 (12.5%) | 2 (28.6%) | 1 (12.5%) | 0 (0%) |
| Study type, n (%) |  |  |  |  |
| Retrospective cohort | 6 (75%) | 5 (71.4%) | 7 (87.5%) | 4 (57.1%) |
| Secondary analysis of prospective cohort study designed for another reason WITHOUT published methodology paper | 1 (12.5%) | 0 (0%) | 0 (0%) | 0 (0%) |
| Prospective cohort study designed to develop or validate the model WITHOUT published methodology paper | 1 (12.5%) | 0 (0%) | 1 (12.5%) | 2 (28.6%) |
| Administrative database | 0 (0%) | 1 (14.3%) | 0 (0%) | 1 (14.3%) |
| Other | 0 (0%) | 1 (14.3%) | 0 (0%) | 0 (0%) |
| Number of centers, median (IQR) | 1 (1, 3.5) | 1 (1, 3) | 1.5 (1, 2.5) | 3 (1, 7) |
| AUC, median (IQR) | 0.9 (0.8, 0.9) | 0.9 (0.8, 1) | 0.9 (0.9, 1) | 0.9 (0.8, 1) |
| Outcome, n (%) |  |  |  |  |
| Infection | 0 (0%) | 0 (0%) | 0 (0%) | 1 (14.3%) |
| Intensive care unit admission | 1 (12.5%) | 0 (0%) | 0 (0%) | 2 (28.6%) |
| Length of stay | 0 (0%) | 0 (0%) | 0 (0%) | 1 (14.3%) |
| Mortality (all-cause, acute pancreatitis specific, does not specify) | 1 (12.5%) | 2 (28.6%) | 0 (0%) | 3 (42.9%) |
| Multisystem Organ Dysfunction/Failure | 2 (25%) | 1 (14.3%) | 0 (0%) | 0 (0%) |
| New onset diabetes | 0 (0%) | 1 (14.3%) | 0 (0%) | 0 (0%) |
| Pancreatic necrosis | 0 (0%) | 0 (0%) | 0 (0%) | 1 (14.3%) |
| Pancreatic necrosis - infected | 0 (0%) | 0 (0%) | 0 (0%) | 1 (14.3%) |
| Severe pancreatitis | 3 (37.5%) | 0 (0%) | 4 (50%) | 1 (14.3%) |
| Moderately severe AND severe pancreatitis | 2 (25%) | 0 (0%) | 1 (12.5%) | 2 (28.6%) |
| Recurrent pancreatitis | 0 (0%) | 0 (0%) | 0 (0%) | 1 (14.3%) |
| Other | 0 (0%) | 3 (42.9%) | 3 (37.5%) | 2 (28.6%) |
| Risk of bias |  |  |  |  |
| Participants |  |  |  |  |
| Low | 2 (25%) | 0 (0%) | 1 (12.5%) | 0 (0%) |
| High | 6 (75%) | 7 (100%) | 7 (87.5%) | 6 (85.7%) |
| Unclear | 0 (0%) | 0 (0%) | 0 (0%) | 1 (14.3%) |
| Predictors |  |  |  |  |
| Low | 2 (25%) | 2 (28.6%) | 0 (0%) | 4 (57.1%) |
| High | 4 (50%) | 5 (71.4%) | 5 (62.5%) | 2 (28.6%) |
| Unclear | 2 (25%) | 0 (0%) | 3 (37.5%) | 1 (14.3%) |
| Outcomes |  |  |  |  |
| Low | 3 (37.5%) | 2 (28.6%) | 0 (0%) | 1 (14.3%) |
| High | 3 (37.5%) | 4 (57.1%) | 8 (100%) | 5 (71.4%) |
| Unclear | 2 (25%) | 1 (14.3%) | 0 (0%) | 1 (14.3%) |
| Statistical analysis |  |  |  |  |
| Low | 0 (0%) | 0 (0%) | 0 (0%) | 0 (0%) |
| High | 7 (87.5%) | 7 (100%) | 7 (87.5%) | 7 (100%) |
| Unclear | 1 (12.5%) | 0 (0%) | 1 (12.5%) | 0 (0%) |
| Overall Risk of bias |  |  |  |  |
| Low | 0 (0%) | 0 (0%) | 0 (0%) | 0 (0%) |
| High | 8 (100%) | 7 (100%) | 8 (100%) | 7 (100%) |
| Unclear | 0 (0%) | 0 (0%) | 0 (0%) | 0 (0%) |

Fidelity = number of items reported/total number of items required (excluding N/A items)
